# Classification of *Andrographis paniculata* extracts by solvent extraction using HPLC fingerprint and chemometric analysis

**DOI:** 10.1186/s13104-020-4920-x

**Published:** 2020-02-04

**Authors:** M. Rafi, A. F. Devi, U. D. Syafitri, R. Heryanto, I. H. Suparto, M. B. Amran, A. Rohman, B. Prajogo, L. W. Lim

**Affiliations:** 1 Department of Chemistry, Faculty of Mathematics and Natural Sciences, Institut Pertanian Bogor, Jalan Tanjung Kampus IPB Dramaga, Bogor, 16680 Indonesia; 2 Tropical Biopharmaca Research Center, Institut of Research and Community Empowerment, Institut Pertanian Bogor, Jalan Taman Kencana No. 3 Kampus IPB Taman Kencana, Bogor, 16128 Indonesia; 3 Department of Statistics, Faculty of Mathematics and Natural Sciences, Institut Pertanian Bogor, Jalan Meranti Kampus IPB Dramaga, Bogor, 16680 Indonesia; 4 Analytical Research Chemistry Group, Institut Teknologi Bandung, Jalan Ganesha No 10, Bandung, 40132 Indonesia; 5 Department of Pharmaceutical Chemistry, Faculty of Pharmacy, Universitas Gadjah Mada, Sekip Utara, Yogyakarta, 55281 Indonesia; 6 Department of Pharmacognosy and Phytochemistry, Faculty of Pharmacy, Universitas Airlangga, Jl Mulyorejo Kampus C Unair, Surabaya, 60286 Indonesia; 7 Department of Chemistry and Biomolecular Science, Faculty of Engineering, Gifu University, 1-1 Yanagido, Gifu, 501-1193 Japan

**Keywords:** *A. paniculata*, Classification, HPLC fingerprint analysis, Principal component analysis

## Abstract

**Objective:**

*Andrographis paniculata*, widely used as an antidiabetic in Indonesian traditional medicines (*jamu*), contains chemical compounds whose concentration is related to its therapeutic effects. The concentration of solvents used for extraction will also affect the number of compounds extracted. Therefore, a quality control method is needed to ensure consistency in quantifying these compounds in *A. paniculata* to improve its therapeutic application. High-performance liquid chromatography fingerprint analysis combined with chemometrics was used to evaluate extracts from different solvent extraction treatments. The content of andrographolide, the main bioactive compound in *A. paniculata*, and the level of α-glucosidase inhibition activity, an indicator of its antidiabetic activity, were also determined.

**Results:**

Fingerprint chromatograms of *A. paniculata* extracts from different treatments exhibited a similar pattern with several peaks in common, only differing in area and intensity value. The *A. paniculata* extracts were classified using HPLC fingerprint and principal component analysis to allow grouping according to their respective solvent extraction treatments. The highest andrographolide content and α-glucosidase inhibition activity occurred in the 50% ethanol extract and the lowest in the water extract. HPLC fingerprint analysis could be used for identifying *A. paniculata* extracts based on solvent extraction, thus improving quality control for their therapeutic application.

## Introduction

*Andrographis paniculata*, commonly known as green chiretta or, in Indonesian, as *sambiloto*, is a medicinal plant often used in Indonesia for treating diabetes. The plant tastes very bitter so is known as ‘king of bitters’. Its major biological activity is antidiabetic [1] but it has also been reported to have anti-angiogenetic [2], antibacterial [3], anti-cancer [4], anti-inflammatory [5, 6], antimalarial [7], antioxidant [8], and hepatoprotective activities [9]. The biological activity of *A. paniculata* comes from its bioactive compounds, the main class being the diterpene lactone group, which includes andrographolide, dehydroandrographolide, neoandrographolide, and deoxyandrographolide. Andrographolide occurs in higher amounts than the other diterpene lactones in *A. paniculata* [10]. Flavonoids such as andrographidine, apigenin, and luteolin are also present in *A. paniculata* [11].

The composition and concentration of chemical compounds in plants are affected by factors such as genetics, environmental growth conditions, and the harvest and post-harvest conditions. The type of solvent used for extraction and its concentration play an important role in the amounts of bioactive compound extracted [12].

Effective quality control of compounds extracted from medicinal plants is needed to ensure the consistency of their biological activity which is related to their content. Two approaches are mainly used for the quality control of medicinal plant extracts: marker and fingerprint analysis [13]. These two approaches have advantages and can be used together to obtain reliable evaluations for the quality control of medicinal plants. Therefore the present study used these two approaches combined with chemometrics to classify extracts from *A. paniculata*.

Several previous studies have reported data on the composition and levels of chemical compounds, and certain biological activities of *A. paniculata* extracts [14–17]. However, the effect of different concentrations of extraction solvent on the levels of marker compounds (andrographolide), chemical fingerprinting and the inhibition of α-glucosidase has not yet been reported for *A. paniculata*. Therefore, the present study aims to investigate the effect of solvent extraction concentration on the chemical compounds extracted from *A. paniculata* using HPLC and the inhibitory effects of these compounds on α-glucosidase.

## Main text

### Materials and methods

#### Materials and chemicals

*Andrographis paniculata* was collected from the *Pusat Studi Biofarmaka Tropika* medicinal plant garden (Bogor, Indonesia) in 2019. Andrographolide (purity > 99.8%) was obtained from ChromaDex Inc. (Santa Ana, CA, USA); ethanol, acetonitrile HPLC grade, and formic acid from Merck (Darmstadt, Germany); Whatman membrane filters (0.22 μm pore size; PTFE; P/N E252, Little Chalfont, UK) for filtration of sample solutions; and alpha-glucosidase and p-nitrophenyl-α-d-glucopyranoside (PNG), phosphate buffer (pH 7), dimethyl sulfoxide (DMSO), and Na_2_CO_3_ from Sigma Aldrich (St Louis, MO, USA).

#### Sample preparation and extraction

The samples of *A. paniculata*, collected 3 months previously, were first sorted then cleaned by washing in water. The samples were then dried and pulverized to a powder. About 10 g of the powder was added to 100 mL of the extraction solvent then soaked with continuous stirring for 6 h then left for a further 12 h without stirring. The solvents used for extraction were water, and solutions of 30%, 50%, 70% and pure ethanol. The filtrate was collected, concentrated with a rotary evaporator, then dried in a freeze-dryer.

#### Determination of α-glucosidase inhibition activity

About 10 mg of *A. paniculata* extract was dissolved using 1 mL DMSO. Fifty µL of 0.1 M phosphate buffer (pH 7), 25 µL of 10 mM PNG and 25 µL of α-glucosidase (0.04 µ/mL) were added to 10 µL of the sample solution followed by incubation for 30 min at 37 °C. The reaction was terminated by adding 100 µL of 0.2 M Na_2_CO_3_. The enzymatic hydrolysis of the substrate to produce p-nitrophenol was monitored at 410 nm using an Epoch microplate spectrophotometer (BioTek Instruments Inc. Winooski, VT, USA). A blank sample and each sample extract were analyzed in triplicate.

#### HPLC conditions

The HPLC conditions were the same as those described by Song et al. [10] with some modifications. An HPLC LC-20 AD equipped with a Shimpack VP ODS C18 column (150 nm × 4.6 mm i.d.) (Shimadzu, Kyoto, Japan) was used to separate the compounds in the *A. paniculata* extracts. The mobile phase used consisted of 0.1% formic acid in acetonitrile (A) and 0.2% formic acid in water (B). The gradient elution was programmed as follows: 10–30% (A) from 0 to 30 min, 30–85% (A) from 30 to 55 min, then 85% (A) from 55 to 60 min. The mobile phase was filtered using a Whatman filter membrane (0.45 µm) and sonicated for 30 min before use. The flow rate was 1 mL/min, and the injection volume 20 μL, with the separation being monitored at 254 nm.

#### Preparation of sample solutions

The sample solutions were prepared by weighing 10 mg of the dried extract, adding 5 mL of 50% methanol (HPLC grade), followed by sonication for 1 h. The sample solutions were then diluted with 10 mL of 50% methanol and filtered through a 0.45-μm membrane filter before injection into the HPLC system. The five different sample solutions (0% as control, 30%, 50%, 70% and pure ethanol) were prepared then injected into the HPLC.

#### Determination of andrographolide content

The andrographolide content of each extract was determined. A series of standard solutions at five concentrations of andrographolide was made in from 10 to 140 μg/mL to construct a calibration curve. The andrographolide content was quantified using the calibration curve with triplicate measurements.

#### Classification of *A. paniculata* extracts

Principal component analysis (PCA) was used to classify the *A. paniculata* extracts. Unscrambler X (version 10.1, CAMO, Oslo, Norway) was used to construct the PCA model using the areas of the eight major peaks from each extract.

### Results and discussion

#### Extraction and inhibition of α-glucosidase activity

The compounds from *A. paniculata* were extracted by maceration at room temperature. The results showed that the extraction yield using different concentrations of ethanol and water solvents differed slightly (Table 1). The highest yield was obtained using 50% ethanol, and the lowest using water, indicating that different concentrations of solvent extraction affected the level of metabolite extracted.

**Table 1:** Effect of solvent concentration on α-glucosidase inhibitory activity and andrographolide yield from *A. paniculata* extracts

Extraction solvent	Inhibition (% ± SD)^a^	Andrographolide content (mg/g ± SD)^b^
Water	54.80 ± 4.05	25.18 ± 1.49
30% ethanol	58.42 ± 2.41	50.29 ± 1.43
50% ethanol	79.66 ± 6.45	114.56 ± 2.30
70% ethanol	60.02 ± 0.32	96.48 ± 0.89
Pure ethanol	49.58 ± 0.97	102.08 ± 2.73

^a^Average of 2 replicates

^b^Average of 3 replicates

The effect of different extraction solvent concentrations on α-glucosidase inhibitory activity was also determined. The assay was based on the principle that α-glucosidase will hydrolyze glucose in the substrate (*p*-nitrophenyl-α-d-glucopyranoside) to α-d-glucose and *p*-nitrophenol so that inhibitory activity can be measured based on the amount of *p*-nitrophenol produced. Table 1 shows that α-glucosidase inhibitory activity was highest in the 50% ethanol extract followed by 70%, 30% ethanol, water and pure ethanol. This result showed that a combination of water and ethanol could extract more polar and semi-polar compounds that are known to have α-glucosidase inhibitory activity.

#### HPLC fingerprint and andrographolide content

**Fig. 1:**
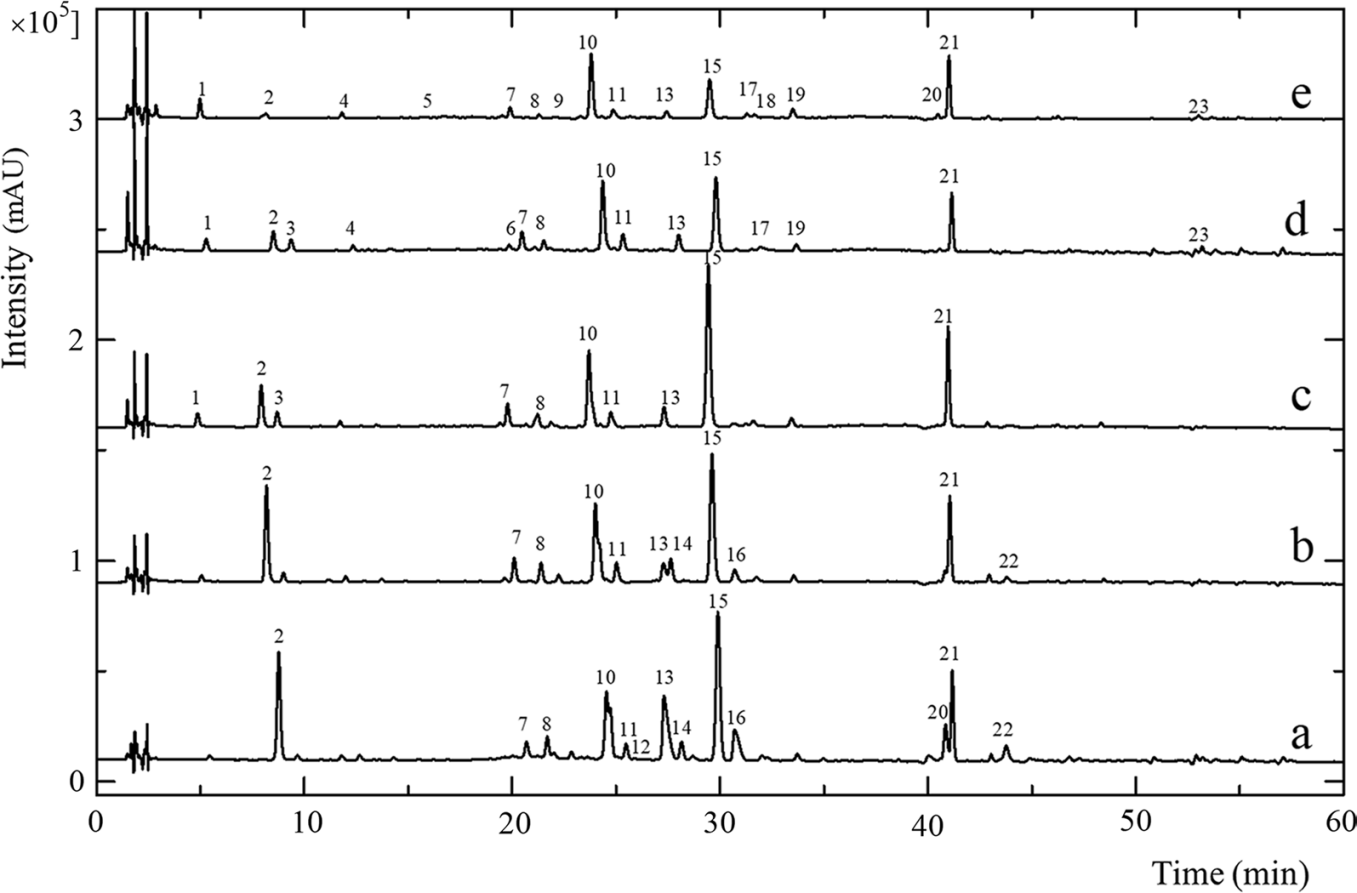
HPLC chromatograms of *A. paniculata* extracts using different solvents: (a) pure ethanol, (b) 70% ethanol, (c) 50% ethanol, (d) 30% ethanol and (e) water

Each extract from *A. paniculata* was analyzed using HPLC to determine the effect of the extraction solvent concentration on the composition of the extracted metabolites. Figure 1 shows the fingerprint chromatogram of the *A. paniculata* extracts. Overall, 23 peaks with a percentage area of more than 5% were detected in the extracts. Peak 15 (andrographolide) was the major peak in *A. paniculata* with the highest intensity and peak area in all extracts. The fingerprint chromatograms obtained of all samples had a similar pattern with peaks 2, 7, 8, 10, 11, 13, 15, and 21 appearing in every sample extract. The differences between the peaks were mostly in peak height and area because each solvent used for extraction exhibited a different polarity and ability for extracting the chemical compounds.

Another difference was that some peaks, such as peaks 12 and 22, appeared only in the ethanol extract thus indicating a typical peak for the fingerprint pattern of ethanol extracts. Peak 1 also appeared in the 30%, 50% ethanol, and water extracts. The extraction solvents with a greater polarity also led to a greater number of detected peaks, the water extracts exhibiting more detected peaks than the other extracts (Fig. 1). These results agreed with a previous study that adding more water to ethanol increased its polarity, thus increasing the yield of diterpenoid lactones [18].

Andrographolide is one of the main bioactive compounds present in *A. paniculata*. The present study determined the andrographolide levels in extracts from five different treatments (Table 1). The highest andrographolide levels were found in the 50% ethanol extract, followed by pure ethanol, 70% ethanol, 30% ethanol, with the lowest in the water extract. These results indicated that the amount of andrographolide extracted depended on the polarity of the extraction solvent. A previous study has shown that andrographolide has a lactone ring which is chemically very vulnerable, reactive and easily rearranged. Opening the lactone ring of andrographolide is the initial stage of the decomposition process. In water, this ring opening occurs through hydrolysis, whereas in ethanol it occurs through a trans-esterification mechanism, with hydrolysis being faster than trans-esterification. Therefore, the rate of andrographolide decomposition depends on the type of solvent. Kumoro et al. [18] reported that adding water leads to the conversion of andrographolide to deoxyandrographolide through the hydrolysis process, thus reducing the andrographolide levels in the extracts.

#### Classification of *A. paniculata* extracts

The HPLC fingerprint chromatograms for the *A. paniculata* extracts used in the present study exhibited a similar pattern, only differing in the peak height and area which corresponded with the level of compound extracted by the different solvent extraction treatments. Differentiating treatments based on HPLC fingerprint chromatograms alone is not easy, so chemometrics analysis is also necessary. Principal component analysis (PCA) can be used to classify or group the extracts according to their solvent extraction treatment. The peak area of the eight major peaks (Peaks 2, 7, 8, 10, 11, 13, 15, and 21) were used as a variable.

Before using PCA, the variable was pretreated by autoscaling. Pretreatment of data is an important step before chemometric analysis to obtain a meaningful result because the quality of the input data greatly affects the quality of the output of the analysis. A common autoscaling method uses the standard deviation as a scaling factor to produce a good analytical output from PCA chemometric analysis techniques [19].

**Fig. 2:**
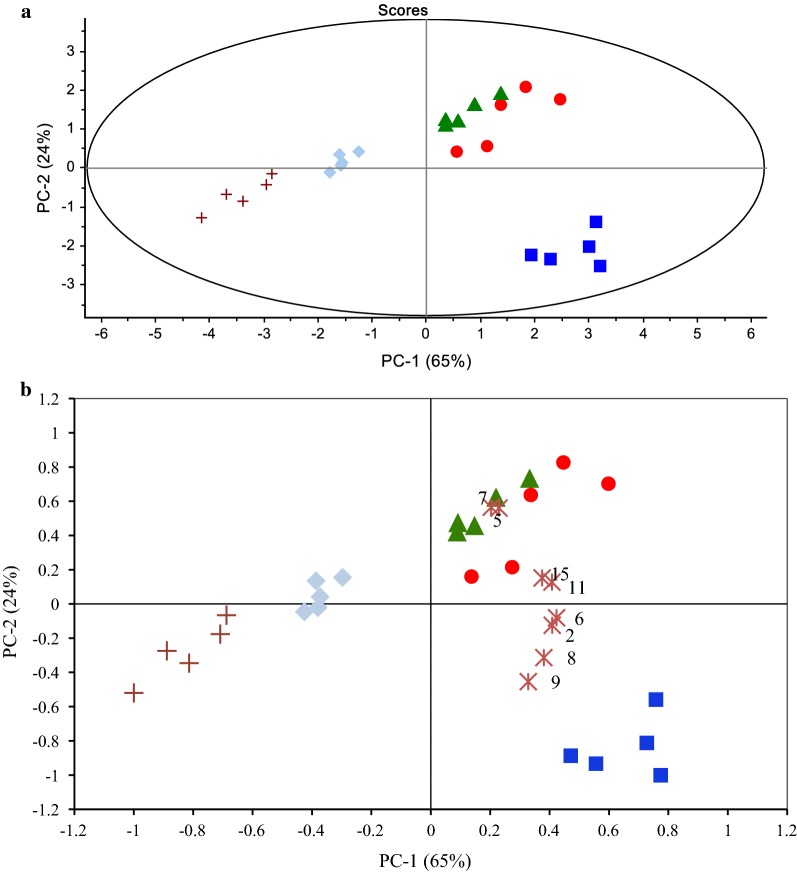
**a** PCA plot and **b** PCA biplot of *A. paniculata* extracts using different solvents: pure ethanol (  ), 70% ethanol (  ), 50% ethanol (  ), 30% ethanol (  ), water (  ), and variable (peak number) (  )

Using PCA, the samples were grouped according to their solvent extraction treatment based on their chemical composition. This multivariate analysis works by simplifying the observed variables by reducing the number of dimensions to give an overview of sample groups using the principal component (PC) [20]. Figure 2a shows the PCA score plot for the *A. paniculata* extracts where the extracts were grouped according to their solvent extraction treatment. Samples with a similar profile of the metabolite will be grouped together and those with a dissimilar profile will form a separate group. The two principal components, PC1 and PC2, explaining most of the variance are used in the analysis. In the present study, the cumulative percentage of the two PCs used was 89% of the total variance. According to Varmuza [21], if the cumulative percentage of PC1 and PC2 is greater than 70%, the score plot offers a good two-dimensional visualization.

The PCA biplot is a combination of the score and loading plots. The loading plot provides information on how strongly each variable affected the principal component. Figure 2b shows the PCA biplot of the *A. paniculata* extracts with the variables which contributed most to its grouping. We found that peaks 5 and 7 contributed strongly to the grouping of the 50% and 70% ethanol extracts of *A. paniculata*.

## Conclusion

The HPLC fingerprint chromatograms of the *A. paniculata* extracts exhibited a similar pattern which differed only in the peak height and area of each detected peak. The 50% ethanol extract provided a higher andrographolide content and percentage α-glucosidase inhibitory activity than the other extracts. Combining the HPLC fingerprint technique with PCA enabled the *A. paniculata* extracts to be classified according to their solvent extraction.

## Limitation

The modified HPLC method was not verified.

## Data Availability

The data can be requested from the corresponding author.

## Abbreviations

DMSO: Dimethyl sulfoxide
HPLC: High-performance liquid chromatography
PCA: Principal component analysis
PC: Principal component
PNG: d-Glucopyranoside
